# Resistin, a Novel Host Defense Peptide of Innate Immunity

**DOI:** 10.3389/fimmu.2021.699807

**Published:** 2021-06-18

**Authors:** Yanran Li, Qiyuan Yang, Dongjie Cai, Hongrui Guo, Jing Fang, Hengmin Cui, Liping Gou, Junliang Deng, Zhisheng Wang, Zhicai Zuo

**Affiliations:** ^1^Key Laboratory of Animal Disease and Human Health of Sichuan Province, College of Veterinary Medicine, Sichuan Agricultural University, Chengdu, China; ^2^Institute of Animal Nutrition, Sichuan Agricultural University, Chengdu, China

**Keywords:** resistin, host defense peptide, anti-bacteria, anti-inflammation, immunomodulation, innate immunity

## Abstract

Resistin, a cysteine-rich protein, expressed in adipocytes, was initially proposed as a link between obesity and diabetes in mice. In humans, resistin is considered to be a pro-inflammatory molecule expressed in immune cells, which plays a regulatory role in many chronic inflammatory diseases, metabolic diseases, infectious diseases, and cancers. However, increasing evidence shows that resistin functions as a host defense peptide of innate immunity, in terms of its wide-spectrum anti-microbial activity, modulation of immunity, and limitation of microbial product-induced inflammation. To date, the understanding of resistin participating in host defense mechanism is still limited. The review aims to summarize current knowledge about the biological properties, functions, and related mechanisms of resistin in host defense, which provides new insights into the pleiotropic biological function of resistin and yields promising strategies for developing new antimicrobial therapeutic agents.

## Introduction

Host defense peptides are endogenous short-peptide compounds that are recognized as a critical component of the innate immune system and are found in epithelial barriers and circulating leukocytes (1, 2). These peptides vary from 10 to 150 amino acids with a net charge between -3 and +20, which have a broad-spectrum antimicrobial activity and diverse immunomodulatory activities (3–6). Resistin (also called ADSF: adipocyte secreted factor; FIZZ3: found in inflammatory zone) is an 11 kDa or 12.5 kDa cysteine-rich secretory protein that consists of 94 and 108 amino acids in mice and humans, respectively. It is a founding member of the resistin-like molecule (RELM) hormone family, which also includes RELMα, RELMβ, and RELMγ (7). The structure of resistin mainly comprises a carboxy-terminal disulfide-rich β-sandwich “head” domain with positive electrostatic surfaces and an amino-terminal α-helical “tail” segment with negative electrostatic potential. Resistin was originally proposed as a novel adipocytokine that is involved in insulin resistance and type 2 diabetes in mice (8–11). In contrast to its production by adipocytes in mice, human resistin is expressed predominantly in leukocytes (12–14), and has always been considered as a pro-inflammatory molecule that plays a regulatory role in many human chronic inflammatory diseases (15), metabolic diseases (16), infectious diseases (17–19), and cancers (20). However, recent studies have shown that resistin can directly kill bacteria by damaging their membranes, revealing that resistin may play a role in antimicrobial innate immunity (21, 22). Also, resistin can limit inflammation caused by microbial products, and modulate numerous host cellular responses (e.g., recruit and activate immune cells, promote the release of proinflammatory cytokines, reinforce interferon (IFN) expression, and promote neutrophil extracellular trap nets (NETs) formation) (23–25). All of these functions favor direct or indirect resolution of infection and reverse potentially harmful inflammation. Therefore, it is becoming significant and necessary to understand the multiple functions and molecular mechanisms of resistin in host defense. The purpose of this review is to provide an overview of the current understanding of resistin as a host defense peptide, with special emphasis on the structure, expression pattern and regulation of resistin, as well as its role and mechanism in host defense. It will provide a new insight into the pleiotropic biological function of resistin and yield promising strategies for developing new antimicrobial therapeutic agents.

## Structure of Resistin

Resistin belongs to the RELM family, which is characterized by a highly conserved, cysteine-rich C terminus and shares structural and sequence homology but exhibits significant diversity in expression within their mammalian host (7). RELMα (FIZZ1) is present mainly in adipose tissue and is highly expressed by airway epithelial cells and type 2 pneumocytes during murine allergic pulmonary inflammation (26, 27). RELMβ (FIZZ2) is specifically expressed by goblet cells and epithelial cells mainly in gastrointestinal tract, however, expression by lung epithelial cells in asthmatic patients and mouse models of lung fibrosis is also observed (27, 28). RELMγ is most highly expressed in the murine hematopoietic system, with high expression in the bone marrow, and lower expression in white blood cells, spleen and thymus (29).

**Figure 1:**
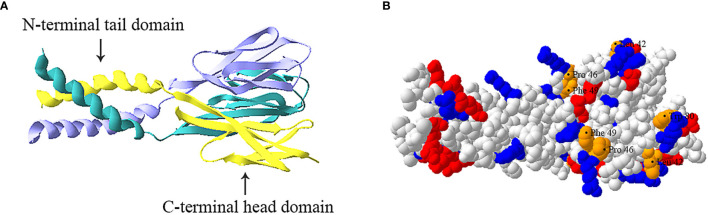
Trimeric structure of human resistin. **(A)** Ribbon model of resistin trimer showing a C-terminal globular domain containing antiparallel β-sheets and an N-terminal α-helical domain. The three chains are indicated in cyan, yellow and lavender respectively. **(B)** Space-filled model of human resistin trimer. The basic (positively charged) amino acids, acidic (negatively charged) amino acids, and amino acid residues (Leu42, Pro46, Phe49, and Trp80) constituting the hydrophobic surface of the “head” region are shown in blue, red, and orange respectively. The structure was predicted based on the structure of mouse resistin structure (Protein Data Bank ID code 1RFX) and constructed by using the SWISS-MODEL program (https://swissmodel.expasy.org).

Resistin is a cysteine-rich polypeptide hormone protein that the mouse and human resistin share 46.7% similarity at the genomic DNA level, 64.4% sequence homology at the mRNA level, and 59% identity at the amino acid level (30). The mouse resistin gene is located on chromosome 8A1 and encodes an 11 kDa cysteine-rich polypeptide that consists of 94 amino acids (11). The human resistin gene located on chromosome 19p13.3 encodes a 12.5 kDa cysteine-rich protein that consists of 108 amino acids (30). These polypeptides both have three main domains: an amino (N) terminal signal sequence, a variable middle section, and a conserved carboxyl (C) terminal that determines the signature of the molecule (30–32). Additionally, the protomer comprises a carboxy-terminal disulfide-rich β-sandwich “head” domain and an amino-terminal α-helical “tail” segment (**Figure 1A**) (33, 34). This carboxy-terminal “head” domain adopts a six-stranded jelly-roll topology and contains two three-stranded all-antiparallel sheets (33, 34). Three protomers associate through the formation of a parallel coiled-coil. Two trimers are further interlinked to form tail–tail hexamers, linking each protomer from one trimer to a protomer from the associated trimer, and forming a short antiparallel six-helix bundle (33, 34).

At present, the basic functions of different domains of resistin have not been clearly recognized. For instance, the C-terminal globular head domain of resistin is proposed to constitute its protein interaction domain (i.e., the binding of resistin to Toll-like receptor 4 (TLR4) and adenylyl cyclase-associated protein 1 CAP1 (35, 36). However, Jang et al. (24) suggest human resistin binds TLR4 mainly through its N-terminal helix. Further studies are needed to confirm it. Besides, the resistin complex exhibits positive electrostatic surfaces in its C-terminal “head” region and negative electrostatic potential in its N-terminal coiled-coil domain (**Figure 1B**) (33). The “head” region of human resistin also contains several residues exposed to the molecular surface, namely Leu42, Pro46, Phe49, and Trp80, which have been proved to play a role in imparting surface hydrophobicity to the molecule (**Figure 1B**) (34). Typically, the cationicity and hydrophobicity of the peptides are considered to be two critical elements for their antibacterial action as they are instrumental for binding and insertion into bacterial membranes (2, 37). It is, therefore, speculated that the C-terminal “head” domain of resistin may be the molecular basis of its antibacterial activity. Consistent with this, Propheter et al. (21) indicated that mRELMβ drives membrane permeabilization mainly through its C-terminal β-sheet structure, which shares 60% sequence homology with the C terminus of human resistin at amino acids level. Future studies should further explore how multiple functions of resistin are related to peptide sequences and structures.

Moreover, the analysis of serum samples shows that resistin circulates in different assembly states in mice and humans, and its state determines its biological activity. In mice, resistin was shown to circulate in two distinct assembly states, the predominant high-molecular-mass (HMW) hexamer and the substantially low-molecular-mass (LMW) trimer complex that is unable to form intertrimer disulfide bonds (33). The LMW monomeric form in mice is considered to be more bioactive and potent in terms of hepatic insulin action impairment (33). In humans, resistin circulates as an oligomer with a molecular mass > 660 kDa and a trimer of 45 kDa (38). In contrast to mice, the oligomeric form of human resistin shows a more potent effect on the stimulation of proinflammatory cytokines (39, 40). As the concentration of the protein was increased, human resistin tends to form oligomers by the formation of inter/intramolecular disulfide linkages, and that it undergoes a concentration-dependent conformational change in secondary structure from α-helical to β-sheet form (41). This switch can be simply attributed to the hydrophobic effect involving shielding the hydrophobic face from the bulk medium (39). Additionally, studies have shown that the structural conformation of resistin may be involved in maintaining the very fine balance of various physiological and pathological conditions (39).

## Resistin Expression Pattern and Regulation

The resistin has been found to express in various organs/tissues, including adrenal gland, pituitary gland, hypothalamus, white adipose tissue, lung, spleen, intestinal epithelium, placenta, pancreas, stomach, skeletal muscle, skin, and plasma (22, 42). Notably, its cell sources and tissue distribution are different among different species. For example, the mouse resistin is almost exclusively expressed in adipocytes of white adipose tissue and is produced during adipocyte differentiation (42). While the human resistin is expressed at very low levels, if at all, in adipocytes, whereas high levels are expressed in leukocytes, including monocytes, macrophages, and neutrophils (13, 43). Moreover, recent studies showed that the human resistin is also expressed in keratinocytes and sebaceous glands within the skin epidermis (22, 44).

Resistin synthesis and release are differentially regulated, depending on the cell type and the microenvironmental stimuli. In human monocytes/macrophages, resistin expression is up-regulated during monocytes differentiation to macrophages (12, 45), indicating a role for resistin in monocyte/macrophage function. It has been demonstrated that proinflammatory mediators such as LPS, tumor necrosis factor-alpha (TNF-α), interleukin 1β (IL-1β), and IL-6 can strongly induce the expression of resistin in monocytes/macrophages (12, 46–48). For instance, *Actinobacillus pleuropneumoniae-*LPS induces porcine alveolar macrophages to produce resistin *via* MyD88, TRAM, and NF-κB by interacting with TLR4 (49). Besides, the mRNA expression of resistin is also significantly upregulated in human peripheral blood-derived monocytes following exposure to microfilariae or antigen of *Brugia malayi* (50, 51). Unlike in monocytes, resistin is stored in two pools after translation inside neutrophils, the one on the membrane of neutrophils, the other in azurophilic granules and specific granules (14). The membrane-attached resistin is up-regulated following stimulation of neutrophils with small formyl peptide derivates (fMLF) and TNF-α. While resistin stored in neutrophils granules is rapidly released when neutrophils are degranulated under the stimulation of fMLF but not TNFα (14). Moreover, some bacteria or selected bacterial components can also induce neutrophils to release resistin (52–57). For instance, *Aggregatibacter actinomycetemcomitans*-expressed leukotoxin can induce the extracellular release of resistin by interacting with LFA-1 on surface of neutrophils and activating Src family tyrosine kinases (57). High levels of *Porphyromonas gingivalis*-LPS trigger resistin release from human neutrophils through PI3K, JNK, and p38 MAPK (54). Additionally, the surface attachment protein of Streptococcus (e.g., M- or M like- protein) can also trigger resistin release from neutrophils. This occurs when soluble M1 protein forms complexes with fibrinogen, which are potent activators of neutrophils through interaction with β_2_-integrins (52, 56). Besides, resistin is also expressed at a low level in human T and B cells but significantly increased in B cells when induced by TNF-α (13).

By contrast, the expression of resistin in sebocytes is not only induced by microorganisms and pro-inflammatory cytokines, but also regulated by vitamin A and its derivatives (22, 44). Vitamin A is a lipid-soluble nutrient that is essential for immunity to infection at many body sites (58, 59). Vitamin A usually regulates gene transcription through its derivative retinoic acid, which binds to retinoic acid receptors (RARs) (60, 61). Harris et al. (22) reported that expression of the human *RETN* gene in sebocytes is enhanced by the vitamin A derivative retinol through direct binding of RARs to the *RETN* promoter, revealing that the expression of resistin in human skin may be related to vitamin-A-dependent innate immunity. Notably, the expression of resistin is significantly up-regulated only when the IL-1β and retinol stimulate human sebocytes, whereas it is not or only slightly up-regulated when retinol or IL-1β was used alone to stimulate human sebocytes (22). This indicates that retinol acts synergistically with a proinflammatory stimulus to stimulate human resistin expression in sebocytes. However, Felipe et al. (62) reported that in mice adipocytes, retinoids could inhibit the expression of resistin, suggesting that the regulation of resistin by vitamin-A may be cell type-dependent or specie-dependent.

## Direct Microbicidal Function of Resistin

Surface tissues of the body (e.g., skin, intestinal tract, and lung) are interfaces with the outside environment and are thus continuously exposed to a diverse collection of microorganisms (63). To cope with the substantial microbial exposure, a diverse arsenal of AMPs, such as defensins, cathelicidins, and C-type lectins, are produced by epithelial surfaces to directly kill or inhibit the growth of microorganisms (64–66). Recent studies indicated that resistin is a bactericidal protein expressed in keratinocytes and sebaceous glands of human skin (22). Moreover, the other RELM family members (i.e., mouse and human RELMβ and mouse RELMα), have also been identified as antimicrobial proteins expressed in intestinal epithelial cells and skin keratinocytes, respectively, which shape resident body surfaces bacterial communities and limit pathogenic bacterial infection of the body surfaces (21, 22). Thus, it is speculated that the RELM family may constitute a previously unknown group of epithelial AMPs that participate in the innate immunity of skin and intestinal mucosa. Here, we make a detailed illustration of the antibacterial functions and action mechanism of resistin or other RELMs *in vitro* and *in vivo*.

### Antimicrobial Activity *In Vitro*

In vitro, human resistin displays a broad-spectrum activity against a variety of Gram-positive and Gram-negative bacteria. For instance, recombinant human resistin was shown to cause a dose-dependent reduction in the viability of strains of the Gram-positive species *Streptococcus pyogenes* and *Propionibacterium acnes* and the Gram-negative species *Pseudomonas aeruginosa*, *Citrobacter rodentium*, *Listeria monocytogenes*, and *Escherichia coli* K12 (21, 22). It is effective microbicides even at concentrations in the range of 2.5-10 μM, and this bactericidal activity functions mainly in the logarithmic phase rather than in the stationary phase of the bacteria (21, 22). Strikingly, this bactericidal activity requires low salt concentrations and an acidic pH (21, 22). This is consistent with the salt and pH sensitivity of other AMPs present on the body surface (67) and may reflect the fact that these bactericidal proteins have evolved to function under the physiological environment of the body. The requirement for these specialized conditions might also explain why resistin was previously reported to lack antibacterial activity (68).

### Antibacterial Function *In Vivo*

**Figure 2:**
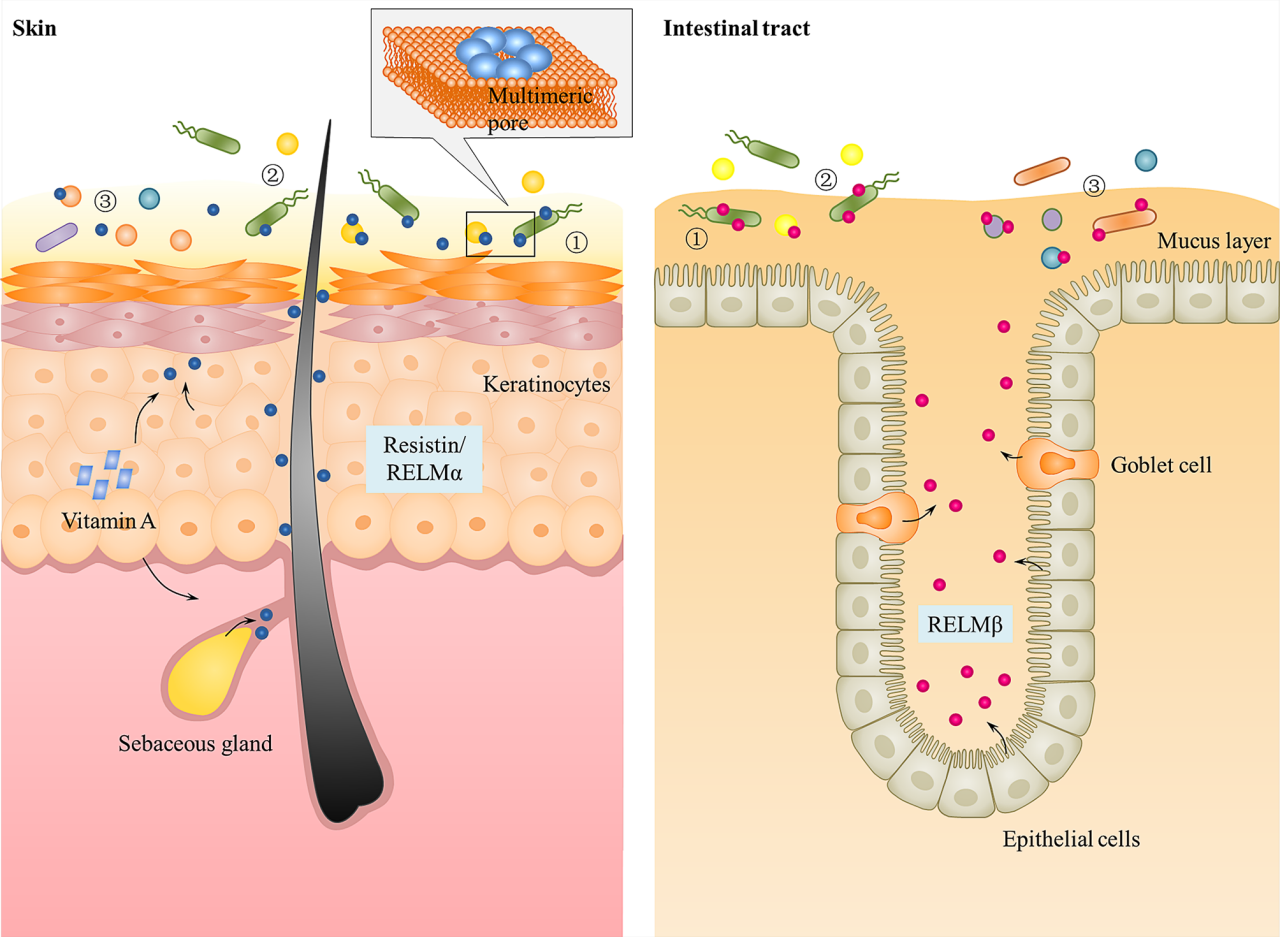
Function of resistin and the other RELMs in skin and intestinal epithelium. Human resistin and mouse RELMα are expressed in keratinocytes and sebaceous glands of skin that can be triggered by bacterial colonization and Vitamin A. Mouse and human RELMβ are expressed by goblet cells and epithelial cells into gastrointestinal tract mucus layer. They kill bacteria by binding to bacterial lipids and forming multimeric pores in bacterial membranes. All RELMs not only ① protect against pathogen colonization, but also ② limit access of the microbiota to host tissues and ③ determine microbiota composition.

Mutant mouse studies have provided effective evidence for the antibacterial function of epithelial RELM family *in vivo* and how they protect against pathogens at the skin and gut mucosae. Due to the lack of human resistin in mice, only mutant mouse models with RELMα gene deletion (*Retnla^−/−^*) or RELMβ gene deletion (*Retnlb^−/−^*) have been constructed. *Retnla^−/−^* mice either intradermally or superficially infected with *S. pyogenes* showed elevated numbers of *S. pyogenes* when compared to wild-type mice (22). Consistent with this, clearance of *C. rodentium* in the colons of orally infected mice is considerably diminished in *Retnlb^−/−^* mice compared with wild-type mice (21). In each of these mutant models, there was a correlation between the antibacterial activity of the RELMs *in vitro* and their ability to confer immunity *in vivo*. Besides, there was a significant two-log increase in the numbers of colonic tissue-associated bacteria in *Retnlb^−/−^* compared with wild-type mice. However, these differences did not extend to the colonic luminal bacterial communities, which were at similar amounts between *Retnlb^−/−^* mice and wild-type mice (21). This suggests that RELMβ can limit the association of bacteria with colonic tissues. Moreover, both *Retnlb^−/−^* mice and *Retnla^−/−^* mice exhibited an altered resident microbiota on the surface of colon tissue or skin (21, 22). Therefore, these investigations show that the RELMs as antibacterial peptides on the skin or intestinal surface not only protect against pathogen colonization but also limit access of the microbiota to host tissues and determine microbiota composition (**Figure 2**).

In addition to skin, the lung is another important source of resistin (12). In the porcine model of pulmonary infection with *Actinobacillus pleuropneumoniae*, the expression of resistin was significantly up-regulated in the porcine lung and hilar lymph nodes, indicating that resistin may play a role in the anti-infection immune response of lung (69, 70). At present, studies only show that resistin(in humans and pigs but not in mice) have the ability to modulate lung cellular responses, including promoting immune cells recruitment, proinflammatory cytokines expression, and the NETs formation, which may indirectly resist pathogen invasion (17, 23, 71). However, whether resistin can play a direct bactericidal role as a bactericidal protein in the lung is still unclear, and further research is needed to confirm.

### Mechanisms of Antibacterial Activity

How do AMPs exert their antimicrobial activity? Many hypotheses have been put forward, including: (1) fatal depolarization of the normally energized bacterial membrane (72); (2) the formation of physical holes that cause cellular contents to leak out (73); (3) the activation of fatal processes such as induction of hydrolases that degrade the cell wall (74); (4) the scrambling of the usual distribution of lipids between the leaflets of the bilayer, resulting in disturbance of membrane functions (75); (5) and the damaging of critical intracellular targets after internalization of the peptide (76). Through transmission electron microscope observation and liposome disruption assays, Propheter and Harris et al. (21, 22) revealed that resistin and the other RELMs are membrane-permeabilizing proteins that cause bacterial cytoplasmic leakage. The mechanism is that the RELM proteins bind to bacterial lipids and form multimeric pores in membranes through their C-terminal, thus lysing the targeted bacterial cells (**Figure 2**). In view of this, a question immediately arises as to how these RELM proteins and other cationic AMPs can function *in vivo* but are not toxic to host cells. One explanation is that unlike bacterial cell membranes, there is a high proportion of cholesterol in mammalian cell membranes. The presence of cholesterol in the target membrane in general reduces the activity of AMPs, due either to stabilization of the lipid bilayer or to interactions between cholesterol and the peptide (4, 77). Consistent with this, the RELMα was shown to be significantly less disruptive to liposomes containing 30% cholesterol than cholesterol-free liposomes (22). Besides cholesterol, the significant differences in lipid compositional and membrane potential between the prokaryotic and eukaryotic membranes also contribute to cell selectivity of cationic AMPs. The outer surface of Gram negative bacteria is covered in lipopolysaccharides and Gram positive bacteria present a surface of teichoic acids, resulting in both classes of bacteria having negatively charged cell surfaces (78). In contrast, most mammalian cell membranes have an outer leaflet comprised of zwitterionic phosphotidylcholine and sphingomyelin phospholipids, whilst the inner leaflet is composed of phosphatidylserine leading to an essentially neutral surface (79). Therefore, the positively charged AMPs may primarily bind to and permeabilize the negatively charged bacterial membrane rather than that of mammalian cells (75, 80). Consistent with this, Propheter et al. (21) found that RELMβ binds to lipids bearing negatively charged lipid head groups (phosphatidylserine and phosphatidic acid), but not to zwitterionic or neutral lipids. And in liposome disruption assays, RELMβ induced rapid dye efflux from liposomes composed of both phosphotidylcholine and phosphatidylserine, while the rate of efflux was reduced when phosphotidylcholine-only liposomes were used (21), which further proves this view. Liposomes composed of phosphatidylserine alone also yielded a reduced rate of dye efflux, suggesting that charge density is an important factor for the RELMs membrane-disrupting activity, a characteristic shared with other cationic AMPs.

Currently, several different models (i.e., the barrel-stave, carpet, detergent, toroidal pore, and aggregate models) have been proposed to explain how, following initial attachment, antibacterial peptides insert into the bacterial membrane to form transmembrane pores which result in membrane permeabilization (75, 80). Nonetheless, the mechanisms utilized by the RELM family have not been as well studied.

## Resistin Molecular Functions on Its Receptors

Up till now, there have been many reports about resistin receptors (e.g., TLR4 (36), CAP1 (35), G-protein-coupled receptors (GPCRs) (81), an isoform of decorin (ΔDCN) (82), and receptor tyrosine kinase-like orphan receptor 1 (ROR1) (83), but no study can powerfully prove the existence of specific receptor for resistin. Resistin exhibits differential binding affinity towards these receptors in different tissues and cell types, thus leading to the activation of diverse cell signaling pathways. Among them, TLR4 and CAP1 serve as the most studied *in vivo* functional receptors for resistin.

### TLR4

**Figure 3:**
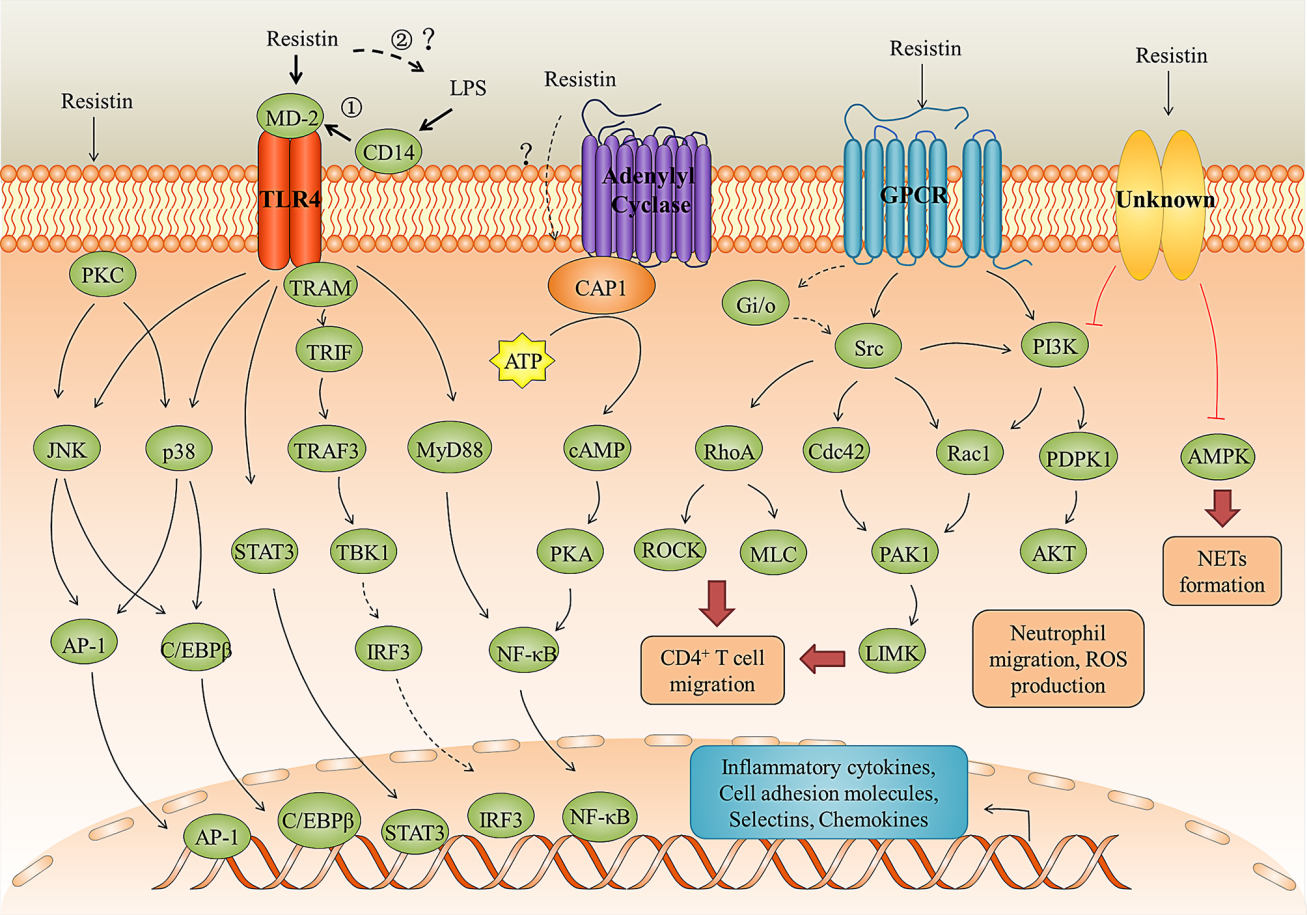
Resistin signaling pathways in inflammation and immune regulation. Resistin binding to the TLR4, CAP1 receptors, GPCR, and unknown receptors activates several signaling pathways leading to the immune cell activation and migration, neutrophil dysfunction, pro-inflammatory cytokines and chemokines production, and NETs formation. The anti-LPS-induced inflammation mediated by resistin through TLR4 may be in two ways: ① Resistin competes with LPS/MD2 for binding to TLR4 and suppresses pro-inflammatory signaling (i. e., NF-κB) while promotes anti-inflammatory signaling (i.e., STAT3, TBK1); ② Resistin directly binds to LPS and neutralize its inflammatory (endotoxin) activity.

TLR4 is a cell surface receptor that plays an important role in resistin-induced inflammation. For instance, Tarkowski et al. (36) first demonstrated that resistin can promote the production of IL-1β, IL-6, and TNF-α in human leucocytes, monocytic cell line THP1, and epithelial cells (HEK293) for binding to TLR4 and activating its downstream NF-κB and MAP-kinases signaling pathway. In porcine alveolar macrophages, resistin promoted the production of pro-inflammatory cytokines *via* TLR4/NF-κB-mediated pathway (TLR4, MyD88/TRAM, and NF-κB) (71). In SH-SY5Y human neuroblastoma cells, resistin inhibited autophagy and increased inflammation by binding to TLR4, inhibiting AMPK phosphorylation, and activating the Akt-mTOR signaling pathways (84). In human nucleus pulposus cells, resistin promoted CCL4 expression through Toll-like receptor-4 and activation of the p38-MAPK and NF-κB signaling pathways, and this expression causes infiltration of macrophage (85). Besides, LPS is known as the main ligand for TLR4. However, when co-stimulation of human peripheral blood mononuclear cells (PBMCs) with resistin and LPS, resistin significantly diminished the LPS-induced production of pro-inflammatory cytokines (24, 36), suggesting that resistin is an endogenous inhibitor of LPS interaction with TLR4 rather than a co-factor of such an interaction. Consistent with this, in a *Nippostrongylus brasiliensis* (*Nb*)-infected human resistin gene expressing transgenic (h*RETN*Tg^+^) mice model in which a lethal dose of LPS was injected simultaneously, resistin decreased circulating proinflammatory and Th1 cytokines (e.g., TNFα, interferon-gamma (IFN-γ), IL-6, IL-12, IL-1α, and granulocyte-macrophage colony-stimulating factor (GM-CSF)), and increased anti-inflammatory cytokine IL-10 through TLR4 (24). However, in the model of *Nb*-infected h*RETN*Tg^+^ mice, resistin significantly increased TLR4 signaling, chemokines, and pro-inflammatory cytokines (17). The accepted explanation to date is that resistin competes with LPS/MD2 for binding to TLR4 and suppresses pro-inflammatory signaling (i. e., NF-κB) while promotes anti-inflammatory signaling (i.e., STAT3, TBK1) (24, 36). However, according to the fact that most AMPs can directly bind to LPS and neutralize its inflammatory (endotoxin) activity (77, 86–88), we speculate that this anti-inflammatory property of resistin may also be the result of neutralizing LPS (**Figure 3**). For example, bactericidal/permeability-increasing protein (BPI) binds the lipid A region of LPS with high affinity, thereby preventing its interaction with other LPS-binding molecules, including LBP and CD14 (89). In this scenario, resistin acts as a scavenger for the activating ligand, removing it before it can trigger inflammation through TLR4. Further studies are needed to confirm it.

### CAP1 and Others

CAP1 is an actin-binding protein, which have three main domains: a carboxy-terminal domain that binds to actin, a central proline-rich domain, and an amino-terminal domain of the unknown function (90). Studies have shown that resistin can directly bind to CAP1 and up-regulate cyclic adenosine monophosphate (cAMP) concentrations, protein kinase A (PKA) activity, and the NF-κB -related transcription of inflammatory cytokines in THP-1 cells (35). However, CAP1 is an intracellular receptor with no predicted transmembrane domain. Therefore, it is likely that resistin may need to bind a surface receptor, such as TLR4, for endocytosis and presentation to CAP1 (**Figure 3**) (35, 91). As for GPCRs, studies have shown that the migration of human CD4 positive lymphocytes induced by resistin may involve the activation of pertussis toxin-sensitive G-protein-coupled receptors, but the interaction between them cannot be effectively proved (81). Decorin and ROR1 are receptors for murine resistin and do not seem to mediate inflammatory responses in humans. Daquinag et al. (83) identified ΔDCN as a functional resistin receptor that may regulate WAT expansion in adipocyte progenitors by modulating cell migration and proliferation and adipocyte differentiation. Sánchez-Solana et al. (82) showed that the interaction of murine resistin with ROR1 in 3T3-L1 preadipocytes leads to the activation of extracellular signal-regulated kinases 1 and 2 (ERK1/2). Murine resistin can also modulate adipogenesis and glucose uptake in 3T3-L1 preadipocytes through the ROR1 receptor.

## Immunomodulatory Function of Resistin

In addition to controlling bacterial burden by direct antimicrobial action, evidence suggests that resistin can indirectly resist microbial invasion/infection by modulating host cellular function, such as activating immune cells and enhancing the production of pro-inflammatory cytokines, inhibiting inflammation induced by microbial products, promoting the formation of NETs, acting as chemokines and/or inducing chemokines production, and enhancing the expression of IFN λ. Besides, the presence of resistin can also contribute to a disturbed immune response in certain contexts and diseases, suggesting that resistin may be a bidirectional immunomodulatory molecule. Here, we review some of the important immunomodulatory functions of resistin that are not related to their direct antimicrobial action.

### Inflammatory Regulation Activity

Studies have shown that resistin can act as a proinflammatory cytokine, activating immune cells and promoting the secretion of proinflammatory cytokines. For instance, resistin activated mouse macrophage-like (RAW264.7) cell, human macrophage-like (U-937) cells, THP-1 cells, porcine alveolar macrophages, and PBMCs, and promoted the production of pro-inflammatory cytokines (i.e., IL-6, IL-1β, and TNF-α)via TLR4 or CAP1-mediated signaling pathways (36, 40, 71). It also sensitized neutrophils to LPS stimulation and enhance the production of TNF-α and MIP-2 by inhibiting the activation of AMPK in LPS-induced neutrophils (23). Additionally, Jang et al. (17) reported that resistin upregulated pro-inflammatory gene expression (i.e., IL-6 and TNF-α) from monocytes and neutrophils in h*RETN*Tg^+^ mice *Nb* infection, which promoted a pro-inflammatory cytokine environment and tipped the balance from type 2 to type 1 cytokines. Strikingly, although this inflammatory environment was negatively impact the host immune response to helminths (e.g., delaying parasite expulsion and exacerbating lung inflammation), the genome-wide transcriptional profiling of Nb-infected lung tissue reveals that resistin can up-regulate almost all the gene ontology-defined immune response genes and defense genes (17). Therefore, it is speculated that the existence of resistin may direct the immune response towards combating more fatal bacterial or viral infections in an environment where viral- or bacterial-helminth co-infections are common.

Consistent with this, Jang et al. (24) identified a protective function for human resistin promoted helminth-induced immunomodulation, with increased survival of *Nippostrongylus brasiliensis* (*Nb*)-infected h*RETN*Tg^+^ mice after a fatal LPS dose compared with naive mice or *Nb*-infected h*RETN*Tg^−^ mice. As mentioned before, this protective effect of resistin is related to its ability to block LPS proinflammatory function (24, 36). Besides, resistin can also reduce the inflammatory response induced by lipoteichoic acid (LTA). LTA is a major cell wall component of Gram-positive bacteria, which is considered to be the counterpart of LPS derived from Gram-negative bacteria (92). Studies have shown that resistin suppressed the production of pro-inflammatory cytokines such as IL-6, TNF-a, and IL-12 p40 in human dendritic cells (DCs) when stimulated by LTA from *Staphylococcus aureus* (93), but the exact mechanism is still unknown.

Collectively, all these observations indicate that resistin has an important role in regulating and balancing inflammatory responses to microbes. Resistin itself or in the case of helminths infection can enhance the host inflammatory response, that further refine and amplify the innate immune response. However, in the case of bacterial-helminth co-infections or stimulation of bacterial products (i.e., LPS, LTA), resistin may inhibit the inflammation associated with microbial challenge. Although the central question regarding the mechanisms by which resistin triggers a switch from pro-inflammatory response to anti-inflammatory response is yet to be clarified, the overall capacity of resistin to regulate inflammation in different environments leads to the conclusion that it is not only involved in suppressing uncontrolled microbial growth, but also modifies inflammation, which prevents microbial products from causing excessive inflammatory damage to the host.

### Enhanced Extracellular Bacterial Killing

Intriguingly, resistin has also been suggested to kill microorganisms through another innate immune mechanism known as neutrophil extracellular trap nets (NETs). NETs are extracellular strands of decondensed DNA in complex with histones and granule proteins, which were expelled from dying neutrophils to ensnare and kill microbes (94, 95). Studies have shown that exposure of neutrophils to resistin resulted in enhanced phosphorylation of the NADPH oxidase subunit p-40phox and NETs formation, as well as increased extracellular concentrations of the alarmins HMGB1 and histone 3 in association with NETs (23). Similarly, the increased NET formation was found in the lungs of h*RETN*Tg^+^ mice subjected to LPS instillation (23). However, Bonavia et al. (96) reported that resistin didn’t affect the extracellular bacterial clearance (i.e., neutrophil phagocytosis or extracellular clearance *via* NETs) of *P. aeruginosa* by neutrophilic-differentiated NB4 cells. These contradictory findings may reveal the context-dependence of resistin’s cellular effects.

### Chemotactic Activity

The migration of immune cells to inflamed and infected tissues is a fundamental process of the innate immune response (97, 98). The mechanisms responsible for the selective recruitment of immune cells into tissues (under homeostatic or inflammatory conditions) are thought to involve cytokines that activate the expression of the E-selectin and P-selectin, intercellular cell adhesion molecule-1 (ICAM-1), and vascular cell adhesion molecule-1 (VCAM-1), as well as leukocyte-specific chemoattractant such as chemokines (99). Studies have shown that resistin can indirectly promote the recruitment of effector cells such as neutrophils, monocytes, and macrophages by stimulating selectins, cell adhesion molecules, and chemokines secretion from a variety of cell types (23, 100–102). For instance, resistin up-regulates the expression of chemokines (i.e., CCL2, CCL3, CCL4, CCL5, CXCL1, and CXCL2) *via* transcription factors NF-κB and C/EBPβ in human articular chondrocytes (100). It enhances VCAM-1 expression and monocyte adhesion through the PKC, p38, and JNK signaling pathways in human osteoarthritis synovial fibroblasts (103). In endothelial cells, resistin up-regulates ICAM-1, VCAM-1, fractalkine, and MCP-1 *via* TLR4, JNK/p38, AP-1, and NF-κB dependent pathways or by Gi/o signaling pathway (104–108). Another mechanism by which resistin induces the expression of P-selectin and fractalkine in endothelial cells is the activation of the transcription factor STAT3 and the up-regulation of SOCS3 expression (109).

**Figure 4:**
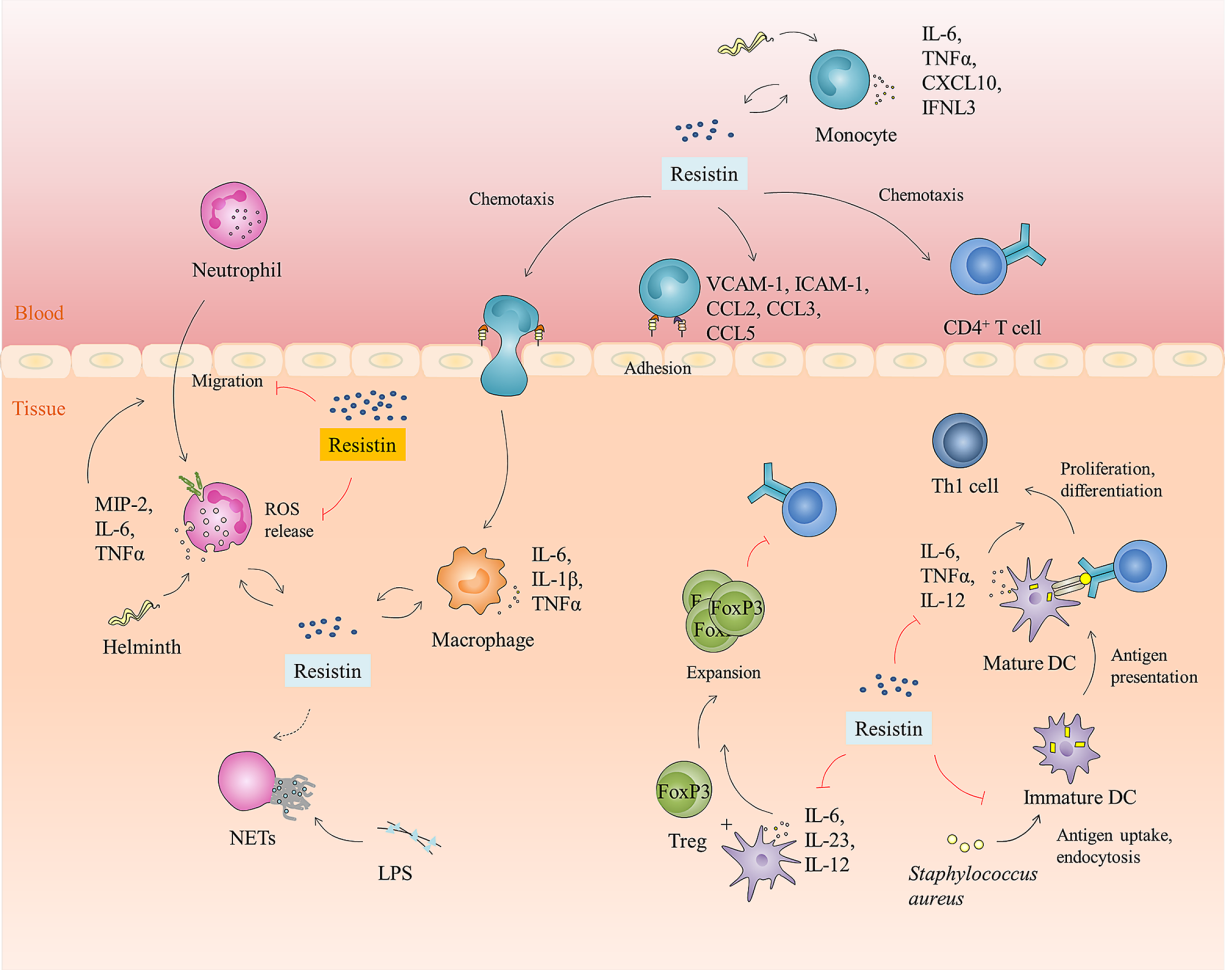
Immunomodulatory function of resistin. Resistin is involved in the host anti-infection immune process by interacting with a variety of immune cell types in humans. It can directly or indirectly promote the adhesion, infiltration, and migration of human monocytes, neutrophils, and CD4^+^ T cells. Likewise, resistin can activate monocytes, macrophages, and neutrophils, promote pro-inflammatory cytokines production, and reinforce IFNL3 expression. Moreover, it can also enhance extracellular bacterial killing, namely promote the formation of NETs. However, in certain diseases or contexts, resistin impedes human neutrophils’ function by inhibiting neutrophils migration and ROS production. It also inhibits antigen uptake, endocytosis, and cytokine production of human DCs, induces expansion of Tregs, and suppresses the proliferation and differentiation of CD4^+^ T cells into Th1 cells by regulating DCs.

Notably, resistin itself also possesses direct chemotactic activity (**Figure 4**). Jang et al. (17) found that *in vivo* treatment of mice with recombinant resistin led to rapid monocyte recruitment that preceded the increased expression of monocyte chemokine signaling genes, including CCR2, CXCL10, suggesting that monocytes can be directly recruited by resistin, and in turn begin to express chemokines as part of a positive feedback loop to promote immune activation. In line with this, resistin showed a direct chemoattractive effect on THP-1 cells and their enhanced migration toward MCP-1 (110). Additionally, resistin also acts directly as a chemoattractant of CD4-positive lymphocytes by binding pertussis toxin-sensitive G-protein-coupled receptors, activating Src-kinase, PI-3 kinase, RhoA, Rac1, Cdc42, PAK, LIMK, ROCK, and phosphorylating MLC (**Figure 3**) (81).

### Enhanced Anti-Viral Immune Response

Furthermore, resistin can also regulate anti-viral immune responses by affecting the expression of interferon. Interferon is a group of low molecular glycoproteins with similar structures and functions produced by host cells when the body is infected with viruses, and it is the most important immune factor against virus infection (111). Studies have shown that resistin can enhance host immunity against HCV infection by reinforcing interferon λ-3(IFNL3)to eliminate hepatitis C virus (25).

### Resistin and Immunosuppression

Studies have shown that patients with type 2 diabetes mellitus, chronic kidney disease, acute kidney injury, *Mycobacterium tuberculosis* infection, and sepsis show increased resistin levels in plasma but also an increased occurrence of infections (68, 96, 112–114). It is mainly because resistin can cause a dose-dependent impairment in neutrophil migration, ROS production, and intracellular bacterial clearance by inhibiting activation of PI3K, PDPK1, and Akt in neutrophils. Notably, this inhibition was observed only when plasma resistin concentrations were >20 ng/ml, but not at normal physiological concentrations (≤20 ng/ml) (**Figure 4**) (96, 114), suggesting that resistin’s cellular effects are context- or disease-dependent. Up to now, the *in vivo* mode of action of resistin on this disturbed immune response has not been clarified. Miller et al. (68) indicated that human resistin can directly impair neutrophil killing of Gram-positive and Gram-negative organisms, while it does not affect the ability of monocytes and macrophages to kill bacteria. However, Nieto et al. (115) reported that resistin can directly reduce the inflammatory phenotype of monocytes and TNF-α production and thus indirectly reduce the oxidative burst of neutrophils.

Besides, when co-cultured with *Staphylococcus aureus*-primed DCs, human resistin can inhibit antigen uptake, endocytosis, and cytokine production (i.e., IL-6, TNF-a, and IL-12 p40) of DCs and thus suppress proliferation and differentiation of CD4^+^ T cells into Th1 cells (93). It can also indirectly induce the expansion of Tregs by inhibiting the expression of IRF-1 and its target cytokines, IL-6, IL-23p19 and IL- 12p40 in DCs when CD4^+^ T cells are co-cultured with DCs (**Figure 4**) (116, 117).

## Perspectives

**Figure 5:**
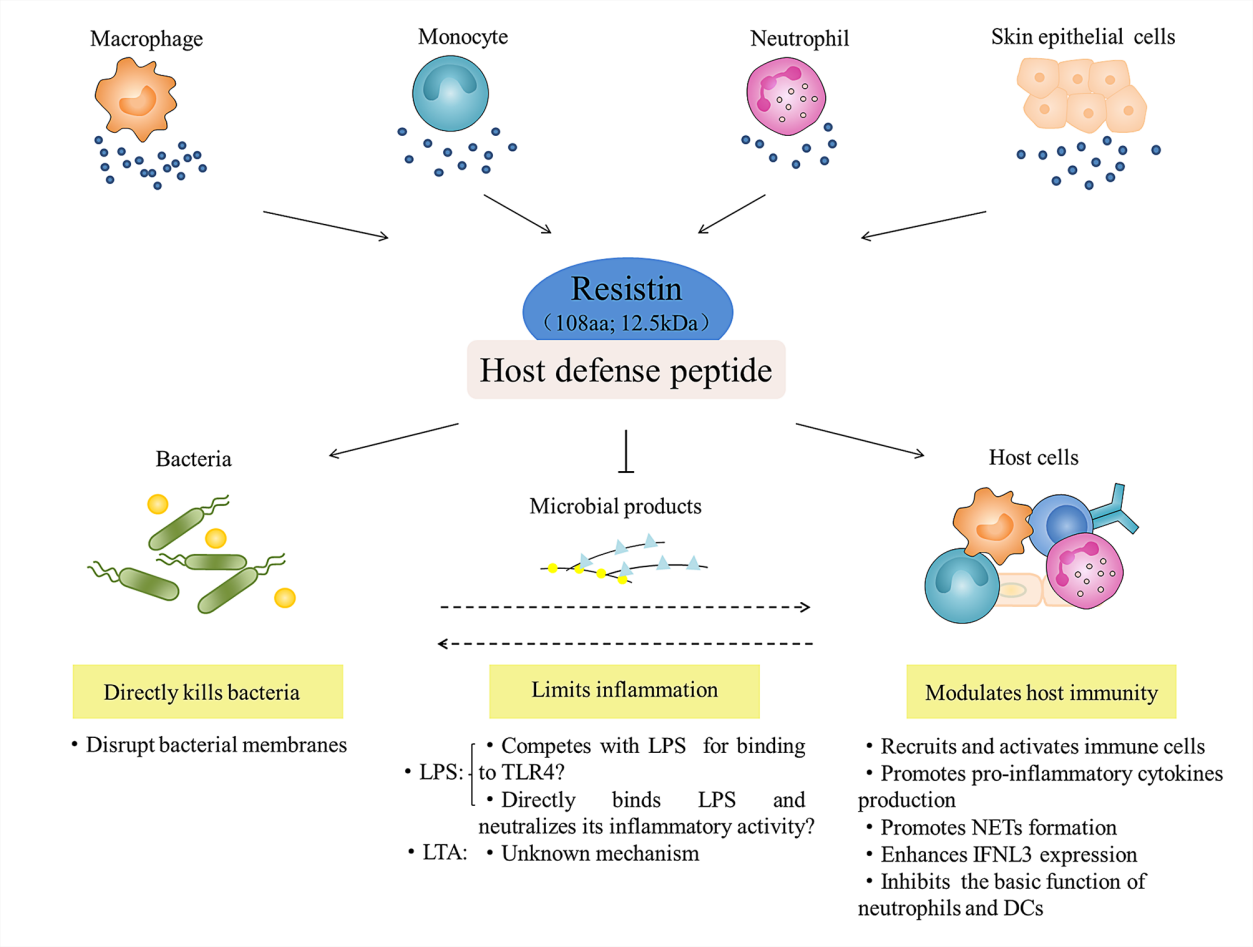
Role of resistin in host defense. Leukocytes (i.e., macrophage, monocyte, and neutrophil) and skin epithelial cells-derived resistin act as host defense peptide, and participate in the innate anti-infection immunity of the host at least three ways: they directly kill bacteria; they regulate and balance the inflammatory response to microbial products (LPS and LTA); and they activate and amplify the host immune response to indirectly resist microbial infection.

In conclusion, as a newly identified host defense peptide (HDP), resistin has the following biological properties (**Figure 5**): (i) it is a 12.5 kDa small molecule protein that consists of 108 amino acids; (ii) it has a positively charged and surface hydrophobic carboxy-terminal β-sandwich “head” domain; (iii) it is mainly expressed in leukocytes and skin epithelial cells in humans; and (iv) it has direct antibacterial, inflammation regulatory, and immunomodulatory functions. Upon exposure to danger, it is becoming clear that resistin is essential not only for inhibiting microbial growth, but also for attenuating exacerbated inflammatory responses and stimulating certain beneficial aspects of inflammation and immunity, which is important for creating an overall balance. Although the current studies have provided effective evidence for the multifaceted biological functions of resistin in host defense, we also find that there are still missing or conflicting information to be solved urgently. For example, many distinct resistin-binding functional receptors have been identified so far, but the specific receptor of resistin and its corresponding signaling pathways need to be further studied. Additionally, when discussing the inflammatory regulation function and related mechanism of resistin, we found that resistin itself, as a pro-inflammatory cytokine, can activate immune cells and promote the secretion of pro-inflammatory cytokines. However, resistin shows anti-inflammatory activity under the condition of bacterial infection or stimulation by bacterial products (LPS). The accepted explanation to date is that resistin competes with LPS/MD2 for binding to TLR4 and suppresses pro-inflammatory signaling (i.e., NF-κB) while promotes anti-inflammatory signaling (i.e., STAT3, TBK1). However, according to the fact that most HDPs have a high affinity for lipopolysaccharide, we propose a hypothesis, that is, whether this anti-inflammatory property is the result of the direct combination of resistin and LPS, thus eliminating LPS before its interaction with TLR4, which is worth further study.

With the growing problem of resistance to and overuse of conventional antibiotics, it is particularly important to explore new antibacterial drugs or new treatment strategies to combat infectious diseases. There is no doubt that HDPs represent the attractive worthwhile candidates to pursue as therapeutic molecules owing to their small size, widespread occurrence among animals and plants, and potent antimicrobial and immunomodulation functions. Therefore, future work should focus on gaining a precise understanding of how resistin acts at the molecular level, that is how the peptide sequence and molecular structure of resistin influence its antibacterial, inflammation and immune regulation functions, which are likely to yield promising strategies for developing new antimicrobial therapeutic agents.

## Author Contributions

YL wrote the manuscript. ZZ, YL, QY, and DC conceived the review and designed the figures. HG and JF helped with the literature and polish the manuscript. HC, LG, JD, and ZW gleaned the material and scrutinized the manuscript. All authors contributed to the article and approved the submitted version.

## Funding

This research was funded by the National Key Research and Development Project (Grant No. 2018YFD0501800), Sichuan Science and Technology Program (Grant No. 2018NZ0002), and the Sichuan Beef Cattle Innovation Team of National Modern Agricultural Industry Technology System (Grant No. SCCXTD-2020-13)

## Conflict of Interest

The authors declare that the research was conducted in the absence of any commercial or financial relationships that could be construed as a potential conflict of interest.
